# Antifungal Activity of Endophytic *Aspergillus terreus* Extract Against Some Fungi Causing Mucormycosis: Ultrastructural Study

**DOI:** 10.1007/s12010-022-03876-x

**Published:** 2022-04-02

**Authors:** Amr H. Hashem, Amr M. Shehabeldine, Amer M. Abdelaziz, Basma H. Amin, Mohamed H. Sharaf

**Affiliations:** 1 Botany and Microbiology Department, Faculty of Science, Al-Azhar University, Cairo, 11884 Egypt; 2 Regional Center for Mycology and Biotechnology, Al-Azhar University, Cairo, Egypt

**Keywords:** Fungal endophytes, Antifungal activity, Mucormycosis, Phytochemical analysis, Ultrastructure study

## Abstract

Endophytes fungi are applied as favorable safe antifungal agents as well as natural bioactive compounds reservoir. In the current study, the inhibitory effect of endophytic fungus was explained by direct antifungal activity against fungi causing mucormycosis, ultrastructural, and determination of active compounds in fungal extract. Endophytic *Aspergillus terreus* was isolated from healthy *Moringa oleifera* leaves and identified morphologically and genetically, and was recorded in gene bank with accession number MW444551.1. Phytochemical analysis and gas chromatography-mass spectroscopy (GC–MS) of ethyl acetate crude extract (EACE) of *A. terreus* were performed. GC–MS results of EACE of *A. terreus* revealed that fungal extract contains 16 major bioactive compounds with extensive pharmaceutical activities. Furthermore, EACE of *A. terreus* revealed a promising antifungal activity against fungi causing mucormycosis as *Rhizopus oryzae*, *Mucor racemosus*, *and Syncephalastrum racemosum*, where inhibition zones of EACE (10 mg/ml) were 20, 37, and 18 mm, respectively. Minimum inhibitory concentration (MIC) of EACE was 0.3125 toward *M. racemosus*, while 1.25 and 2.5 mg/ml against *R. oryzae and S. racemosum*, respectively. In the same context, treated *R. oryzae*, *M. racemosus*, *and S. racemosum* with EACE of *A. terreus* revealed elevation of membrane lipid peroxidation which approves membrane leakage. Furthermore, ultrastructure changes were observed which established alteration in both sporangium and hyphal structures; cell membrane and cytoplasm leakage. In conclusion, endophytic *A. terreus* has an outstanding antifungal activity against fungi causing mucormycosis.

## Introduction

*Moringa oleifera*, considered one of the most important multifunctional plants, is studied for food and medicinal usages, water purification, bio-pesticide, and production of biodiesel [1]. All parts of *Moringa oleifera* such as leaves, pods, and seeds are considered to be one of the healthiest and beneficial foods due to reservoir of bioactive compounds such as flavonoids, phenolics, vitamins, carotenoids, and alkaloids [2]. *Moringa oleifera* is considered a very amusing storehouse source of endophytic fungi [3]. Endophytic fungi have antimicrobial activity in addition to their antioxidant activity through synthesis of range of phenolics, with low cytotoxicity effect against ATCC-CCL-81 cell line [4]. Ethyl acetate extract derivative from endophytic fungi has a great activity against diverse pathogenic microorganisms due to the occurrence of active secondary metabolites including steroids, flavonoids, terpenoids, peptides, quinones, lignans, alkaloids, phenylpropanoids, phenolics, and isocoumarins [4]. Endophytic fungi could produce effective antifungal metabolites which can resolve growing invasive fungal infections [5]. Crude extracts of endophytic *Aspergilli* have promising antibacterial against Gram-positive and Gram-negative bacterial pathogens as well as fungicidal against pathogenic fungi [6]. Endophytic fungi as reservoirs of unique bioactive complexes that have hopeful therapeutic capacities such as antimicrobial and antiviral and may be effective against COVID-19 [7]. The discovery of new bioactive secondary metabolites from endophytic microorganisms including fungi is a vital different way to overcome the growing levels of drugs resistance to many pathogenic fungi [8].

Aggressive fungal infections are considered a major cause of sickness and mortality in immunocompromised patients [9, 10]. Mucormycosis is considered very destructive invasive fungal diseases [11]. Human mucormycoses are caused by a wide range of fungal pathogenic linked to the mucorales including *Rhizopus*, *Mucor*, and *Syncephalastrum*. Mucormycosis occurs typically in immune-compromised patients, and it was hard and difficult to control infections due to imperfect diagnostic tools and therapeutic decisions [12]. The main objective of this article was to isolate and identify endophytic fungi that have the ability to produce biologically active substances thus applied to control fungi causing mucormycoses (The Black fungus) which are accompanied to COVID-19 infections and moreover to detect the ultrastucture changes of fungi as a result of treatment with EACE of the selected fungal endophyte.

## Material and Methods

### Source of Plant Sample

*Moringa oleifera* was obtained kindly from the National Research Center (NRC), Dokki, Egypt (30.036624896199623 N) (31.205301460781154 E). The plant parts were put in plastic bags and transported to the laboratory and stored in a refrigerator at 4 °C for further use.

### Isolation and Identification of Endophytic Fungus

Isolation of established endophytic fungal isolates was assessed by the method of Aldinary et al. [3]. Healthy *M. oleifera* leaves were outer surface washed by tap water, then sterilized in 70% ethyl alcohol for 1 min, then 4% NaOCl for minute soaking, and finally washed three times in sterile distilled water and dried by sterilized filter paper. Sterilized *M. oleifera* leaves were cut into 0.5 × 0.5 cm by sterilized scalpel, then moved to sterilized potato dextrose agar (PDA) medium (Sigma-Aldrich, Germany) supplemented with antibiotic chloramphenicol (0.2 g/L), and then incubated at 27 °C ± 2 for 21 days and daily observed under a stereomicroscope. Then, the endophytic fungal strain was identified morphologically and genetically. Morphological identification of the fungus was carried out with observing the morphological characteristics (color, texture, and appearance) and microscopic characteristics using light microscope [13–16]. DNA was extracted from agar cultures using Quick-DNA Fungal Microprep Kit (Zymo research; D6007) following the manufacturer’s protocol and supported by Sigma Scientific Services Company (Egypt). PCR was performed by using Maxima Hot Start PCR Master Mix (Thermo; K1051). The primers used were forward ITS1-F (5′-TCCGTAGGTGAACCTGCGG-3′) and reverse ITS4-R (5′-TCCTCCGCTTATTGATATGC-3′) according to the method used by Visagie et al. [17] and Khalil et al. [18] and Khalil et al. [4].

### Extraction of Fungal Active Compounds

Fungal isolate was transferred into 1000-ml potato dextrose broth medium (PDB) in 2000-ml Erlenmeyer flasks and kept at 28 °C for 21 days in shaking (150 rpm) incubator and extracted by using ethyl acetate. Filtrate was mixed with equivalent volume of ethyl acetate and placed on a vortex shaker for 10 min and remained for 5 min until the two clear separate layers. The ethyl acetate layer was separated from the aqueous layer by the separating funnel. The collected phase was evaporated using oven at 60 °C. Finally, DMSO at 1 mg/ml of concentration was used to dissolve the fungal crude extract and then stored at − 20 °C until further experiments. Fungal isolate was transferred into 1000-ml PDB in 2000-ml Erlenmeyer flasks and kept at 28 °C for 21 days in shaking (150 rpm) incubator and extracted by using ethyl acetate. Filtrate was mixed with equivalent volume of ethyl acetate and placed on a vortex shaker for 10 min and remaining for 5 min until the two clear separate layers. The ethyl acetate layer was separated from the aqueous layer by the separating funnel. The collected phase was evaporated using oven at 60 °C. Finally, dimethyl sulfoxide (DMSO) at 1 mg/ml of concentration was used to dissolve the fungal crude extract and then stored at − 20 °C until further experiments.

### Qualitative Screening of Phytochemicals

Qualitative screening of phytochemicals (alkaloids, flavonoids, glycoside, steroid, terpenoids, tannin, saponin, and reducing sugar) was performed according to the methods used by Gul et al. [19] and Sarkar et al. [20]

### Gas Chromatography-Mass Spectroscopy (GC–MS) Analysis

The metabolites present in the EACE *A. terreus* was analyzed, counted, and identified using GC–MS as explained by Zothanpuia et al. [21] with minor modifications. Extract was dissolved in spectroscopy-grade methanol. GC–MS analysis was done using Trace GC1310-ISQ mass spectrometer (Thermo Scientific, Austin, TX, USA) with a direct capillary column TG–5MS (30 m × 0.25 mm × 0.25 μm film thickness). The column oven temperature was maintained at 50 °C at the start and risen at a rate of 5 °C/min to 230 °C and then held for 2 min, and subsequently increased to the final temperature of 290 °C and kept for 2 min. The injector and MS transfer line temperatures were held at 250 andd260°C, respectively. The sample (1 µl) was injected at 250 °C utilizing helium as a carrier gas, split at the ratio of 1:30. Mass spectrometer was operated in the electron ionization (EI) mode in 200 °C at 70 eV with a scan range of 40–1000 m/z. The spectrum of the detected compounds was compared with the spectrum of the known compounds stored in the WILEY 09 (Wiley, New York, NY, USA) and NIST 11 (National Institute of Standards and Technology, Gaithersburg, MD, USA) library. The name, molecular weight, and chemical structure of the detected compounds were also determined [22–25].

### Antifungal Activity

The antifungal activity of EACE *A. terreus* was evaluated on PDA medium. The test of agar well diffusion was performed in accordance with the documenttM51-A2 of the Clinical Laboratory Standard Institute [26]. *A. terreus* was initially grown on PDA plates and incubated at 30 °C for 3–5 days [16, 27]. The fungal suspension was prepared in sterilized phosphate buffer solution (PBS) pH 7.0, and then the inoculum was adjusted to 10^7^ spores/ ml after counting in a cell counter chamber. One milliliter was uniformly distributed on agar MEA plates. Wells (6 mm) were cut using a sterile cork borer; 100 µl of EACE was transferred to each well individually and left for 2 h at 4℃. Amphotericin B was used as standard antifungal, and then, the plates were incubated for 3 days at 30℃. After incubation, the inhibition zones were determined and recorded. Moreover, different concentrations of EACE *A. terreus* were evaluated as antifungal to detect minimum inhibitory concentration (MIC).

### Measurement of Membrane Lipid Peroxidation

The membrane lipid peroxidation can be analyzed by measuring the malondialdehyde (MDA) level. The MDA level was measured with the thiobarbituric acid assay [28] Briefly, 10 mm from each tested mycelial discs at the edge of an actively growing colony was inoculateddintoo250-ml flasks containing sterilized PD (100 ml). The inoculated flasks were incubated in a shaker at 28 °C att140 rpm for 48 h. The hyphae were collected, again inoculated into PD and incubated. Then, the active mycelia were respectively inoculated into 0.1 M phosphate buffer solution (PBS, pH 7.2) with ethyl acetate extract at EC 50, and incubated in a shaker at 28 °C at 140 rpm at different times 12, 24, 36, 48, 60, and 72 h, using PBS as a control. The treated hyphae were collected, washed, homogenized, and centrifuged to determine MDA level [29]. The values were the means of three replicates. Each treatment within a replicate was repeated three times. Statistical analysis was carried out estimated by GraphPad Instat software.

### Electron Microscopy

TEM preparation for fungi and fungal specimens (nearly 1mm^3^, each) was removed from agar colonies. The samples were fixed in 3% glutaraldehyde, rinsed in phosphate buffer, and post-fixed in potassium permanganate solution for 5 min at room temperature. The samples were dehydrated in an ethanol series ranging from 10 to 90%ffor 15 min in each alcohol dilution and finally with absolute ethanol forr30 min. Samples were infiltrated with epoxy resin and acetone through a graded series till finally in pure resin. Ultrathin sections were collected on copper grids. Sections were then double stained in uranyl acetate followed by lead citrate. Stained sections were observed with a JEOL-JEM 1010 TEM at 70 kV at RCMB, Al-Azhar University [30, 31].

### Statistical Analysis

Data are presented as means ± SD of at least three independent experiments. Comparisons are made by the Student’s *t*-test or by ANOVA when appropriate. Differences are considered statistically significant at *P* < 0.05. Statistical analysis was carried out estimated by GraphPad Instat software.

## Results and Discussion

### Identification of the Endophytic Fungal Strain

**Fig. 1:**
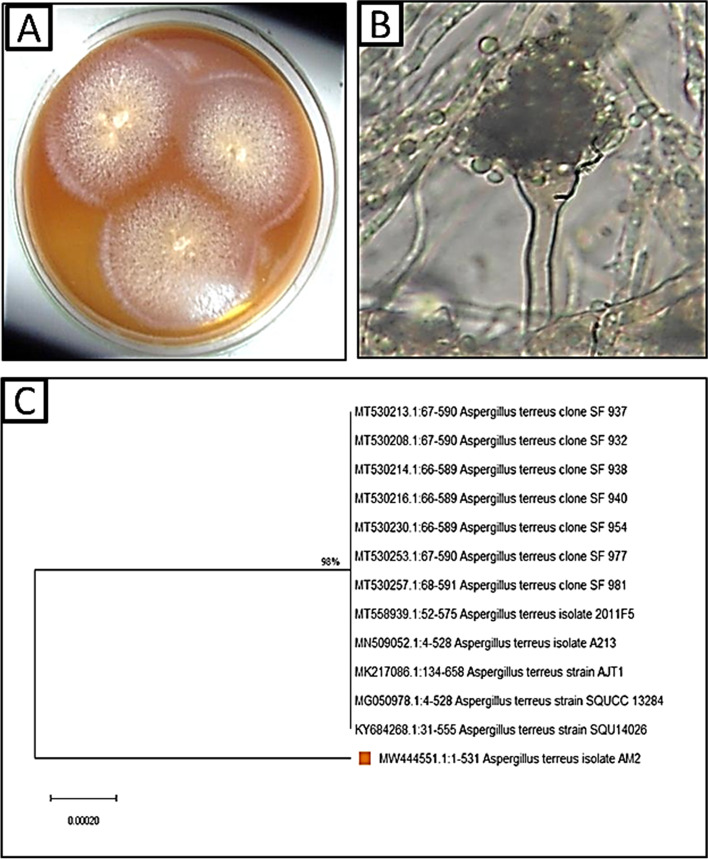
**A** Surface of *Aspergillus terreus*; **B** conidiophore and conidia of *A. terreus*; **C** phylogenetic tree of *A. terreus* isolate AM2

Morphological identification illustrated moderate growth frequency with finely granular conidial creation at 25 ± 2 °C on PDA medium 35 to 60 mm diameter after 7 days. Colonies surface buff to cinnamon with reverse yellow to orange pigments dark in center due to the presence of hyaline cleistothecia, reverse sometimes deeper and with cleistothecia surrounded by gray yellow or buff Hull cells (Fig. 1A). Microscopically, hyaline mycelium is septated, and conidial heads are biseriate, columnar hyaline, conidiophores 70 to 300 µm with smooth-walled, ending with globose vesicles. Pyriform vesicles, 8 to 12 mm wide bearing hyaline conidia, are small (2–2.5 µm), globose, and smooth (Fig. 1B). Molecular identification illustrated the isolated fungal strain AM2 is similar to *Aspergillus terreus* with 98% and recorded in gene bank with accession number MW444551.1 The phylogenetic analysis of fungal strains revealed 98% identity with ITS sequences of rRNA genes of related species using BLAST programs (Fig. 1C).

### Phytochemical Analysis

The existence of secondary metabolites reveals the importance of natural product as therapeutic agents. Fungal extracts are a valuable source for many other secondary metabolites such as antibacterial, antifungal, anticancer, and antiparasitic compounds. The results of preliminary phytochemical study are given in Table 1 which presented some secondary metabolites that are found in EACE *A. terreus*. Glycosides serve as antifungal agent through acts as a specific inhibitor of glucan synthesis in cells and in vitro, and leads to morphological changes in yeasts and molds [32]. Flavonoids often fungal growth with various underlying mechanisms, including plasma membrane disruption and the induction of mitochondrial dysfunction, minimized the following: cell wall formation, cell division, RNA and protein synthesis, and the efflux-mediated pumping system [33]. Terpenoids possess antitumor, anti-inflammatory, antibacterial, antiviral, and antimalarial effects, promote transdermal absorption, prevent and treat cardiovascular diseases, and have hypoglycemic activities [34]. Terpenoid is a cell wall inhibitor class of antimicrobial phytochemicals which the mechanism of action is by membrane disruption [35]

**Table 1:** Phytochemical screening of EACE *A. terreus*

No	Secondary metabolite	Extract
1	Alkaloids	-
2	Glycosides	+
3	Flavonoids	+
4	Steroids	-
5	Terpenoid	+
6	Tannins	-
7	Reducing sugar	-
8	Saponins	-

+ , mean present; -, mean absence

### GC–MS

**Fig. 2:**
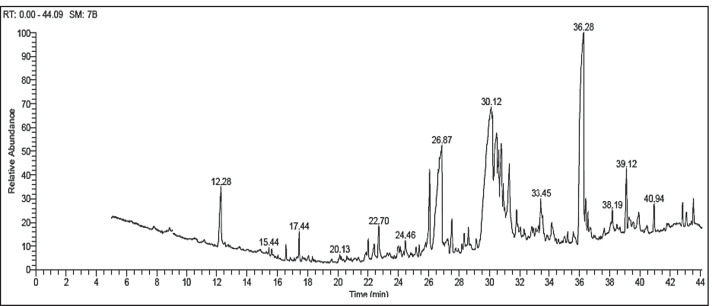
GC–MS chromatogram of ethyl acetate extract of *A. terreus*

GC–MS analysis gives a representative spectral output of each one of the compounds found in the analyzed samples. GC–MS has become well documented as a major technology platform for describing the secondary metabolites in both plant and non-plant species [36]. The results from the analysis of EACE *A. terreus* indicated that the average yield of the extract is about 16 major compounds as shown in Fig. 2 and Table 2. The known compounds were categorized into three groups including major compounds (more than 10%), minor compounds (less than 10% and more than 1%), and trace compounds (less than 1%). Based on this classification, 1,2-Benzenedicarboxylic Acid, Di iso octyl Ester; 9-Octadecenoic Acid,E and Hexadecanoic Acid were major compounds with ratio 26.12, 13.75 and 10.84% respectively. While (Benzaldehyde, 4-Nitro; 10,13-Octadecadiynoic Acid, Methyl Ester; 1,2-Dihydro-4-Methyl-6-Nitro-2-Oxo Quinoline; Pentadecanoic Acid,14-Methyl-, Methyl Ester; 1,2-Benzenedicarboxylic Acid, Butyl Decyl Ester; 2 h-Pyran,Tetrahydro-2-(12-Penta Decynyloxy); 9-Octadecenoic Acid (Z)-, Methyl Ester; Octadecanoic Acid; Androstan-17-One,3-Ethyl-3-Hydroxy; 9,12-Octadecadienoic Acid (Z,Z); Androst-4-En-3-One, 17-Methoxy-, 3-Methoxime, (17á); Eicosanoic Acid; 9-Octadecenoic Acid (Z)-,2,3-Dihydroxypropyl Ester; Octadecanoic Acid,2-Hydroxy-1,3-Propanediyl Ester; Isochiapin B; Pyridine, 2,4,6-Triphenyl; and Ethyl Iso-Allocholate) were minor components with ratio range from 1.23 to 4.18%. In addition to Benzaldehyde, 4-Nitro; Decane, 1-Chloro; 1,4-Benzenediol 2-(1,1-Dimethylethyl)-5-(2-Propenyl); 1-Bromododecane; 1-Hexadecanol, 2-Methyl; Hexadecane-1-Bromo; Tetradecanoic Acid; 1-Tetradecanol; Oleic Acid-Eicosyl Ester; were considered as traces compounds. The antifungal activity of extract may be related to the major and minor compound which have antimicrobial activity such as anti-inflammation, anticancer, hepatoprotective, antihistamine, hypocholesterolemic, antieczemic, antioxidant, hypocholesterolemic, pesticide, nematicide, antiandrogenic, hypocholesterolemic, antiarthritic, nematicide, 5-alpha reductase inhibitor, antiacne, hepatoprotective, antitumor, and antifungal. Most of the identified compounds are related to fatty acids particularly linoleic acid, stearic acid ester, and myristic acid. Different studies reported that these compounds were extracted from the metabolite endophytic fungi living in plants [6, 37], and the occurrence of this compound can make the different biological activity as mentioned above in Table 2 [38].

**Table 2:** The detected compounds through GC–MS of EACE *A. terreus*

No	Compound name	RT (min)	Peak area %	activity	References
1	Benzaldehyde, 4-Nitro-	12.28	2.69	Not reported	-
2	1,2-Dihydro-4-Methyl-6-Nitro-2-Oxo Quinoline	22.7	1.48	Not reported	-
3	1,2-Benzenedicarboxylic Acid, Butyl Decyl Ester	26.07	3.01	No activity reported	-
4	Hexadecanoic Acid	26.86	9.23	Anti-inflammation, anticancer	Kim et al. [46]
5	2 h-Pyran, Tetrahydro-2-(12-Penta Decynyloxy)-	27.53	1.93	No activity reported	-
6	9-Octadecenoic Acid (Z)-, Methyl Ester	28.61	2.14	Antioxidant, antimicrobial, cancer enzyme inhibitors	El-Fayoumy et al. [47]
7	9-Octadecenoic Acid, (E)-	30.12	17.03	Antimicrobial	El-Din and Mohyeldin [40]
8	10,13-Octadecadiynoic Acid, Methyl Ester	30.61	4.01	Antibacterial and antifungal	Agoramoorthy et al. [41]
9	Androstan-17-One, 3-Ethyl-3-Hydroxy-,	30.79	3.41	Anticancer, antimicrobial	El-Far et al. [48]
10	9,12-Octadecadienoic Acid (Z,Z)	31.32	2.69	Hepatoprotective, antihistamine, hypocholesterolemic, anti-eczemic	Chenniappan et al. [49]
11	Androst-4-En-3-One, 17-Methoxy-, 3-Methoxime, (17á)-	32.8	1.81	Antimicrobial	Amiranashvili et al. [50]
12	Eicosanoic Acid	33.45	2.61	No activity reported	-
13	1,2-Benzenedicarboxylic Acid, Diisooctyl Ester	36.26	26.12	Antimicrobial	Rajeswari et al. [51]
14	Pyridine, 2,4,6-Triphenyl-	39.12	2.91	Anticancer	30 [52]
15	Ethyl Iso-Allocholate	39.92	2.59	Antifungal	Abubacker and Devi [39]
16	Isochiapin B	40.94	5.29	Anti-insect and antitumor	Elsharkawy [53]

### Antifungal Activity

**Fig. 3:**
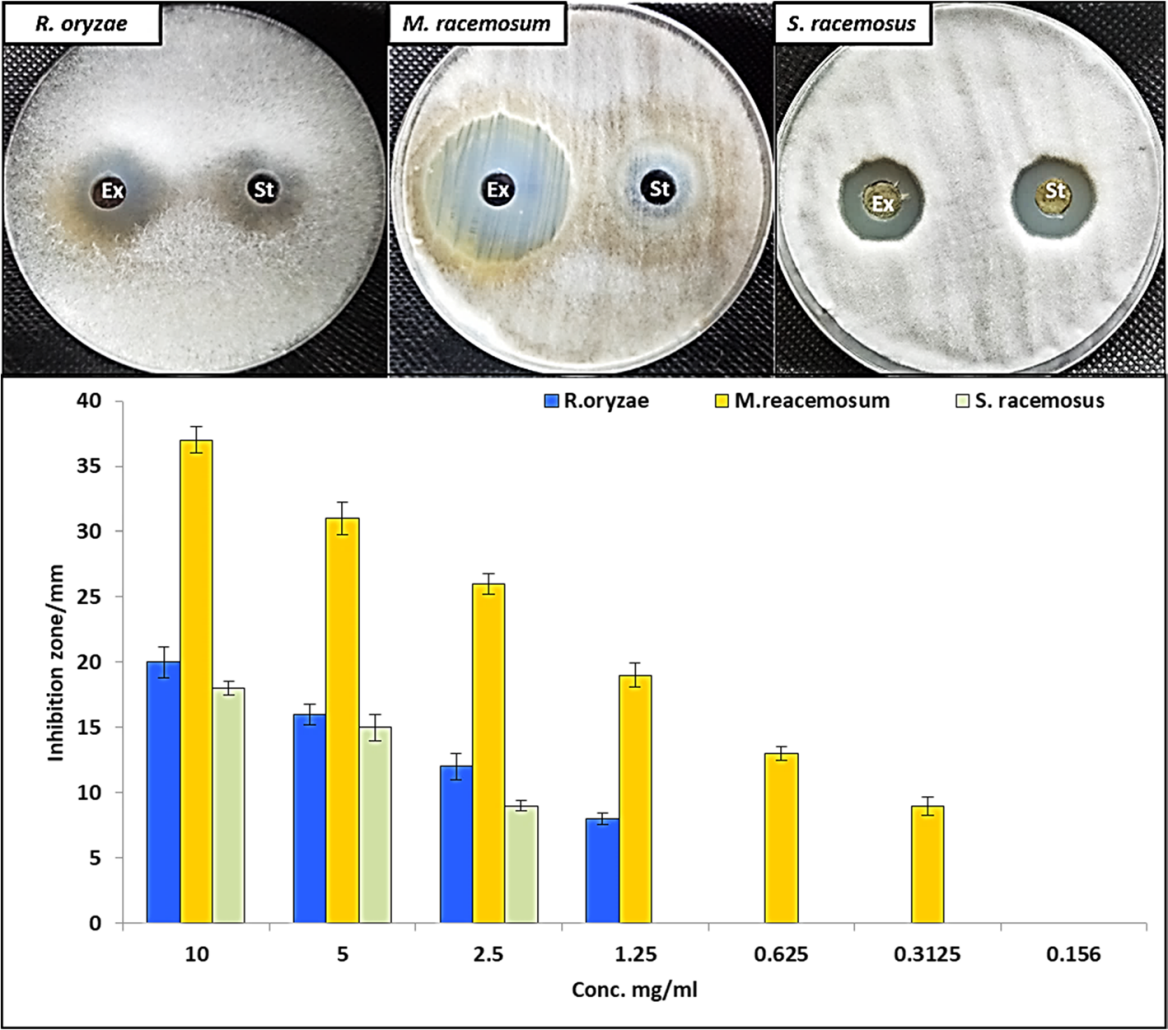
Antifungal activity of EACE of *A. terreus* against *R. oryzae*, *M. racemosus*, *and S. racemosum.* Ex and st mean EACE *A. terreus* and standard antifungal (amphotericin B)

Endophytes are a potent source of bioactive compounds that mimic plant-based metabolites. Numerous bioactive compounds with antimicrobial, anticancer, antioxidant, and immunomodulatory properties are known to be derived from fungal endophytes [4]. Therefore, metabolites of fungal endophytes are used as antimicrobial particularly antifungal to control of resistant microbes. In this study, different concentrations of EACE *A. terreus* were evaluated as antifungals against *R. oryzae*, *M. racemosus*, *and S. racemosum* as shown in Fig. 3. Results revealed that EACE *A. terreus* has potential antifungal activity against fungi causing mucormycosis such as *R. oryzae*, *M. racemosus*, *and S. racemosum*, where inhibition zones at concentration (10 mg/ml) were 20, 37, and 18 mm, respectively. Moreover, MIC was detected through evaluation the antifungal activity of each fungal strains at different concentration (10, 5, 2.5, 1.25. 0.625, 0.3125, and 0.156 mg/ml). Results illustrated that MIC of EACE was the best toward *M. racemosus* where it was 0.3125 mg/ml, while it was 1.25 and 2.5 mg/ml against *R. oryzae and S. racemosum*, respectively, as displayed in Fig. 3. This antifungal activity of EACE is due to the presence of multiple compounds that have antifungal activity as ethyl iso-allocholate [39], 9-octadecenoic acid [40], 10,13-octadecadiynoic acid [41], oleic acid [42], and 1-bromododecane [43].

### Lipid Peroxidation

**Fig. 4:**
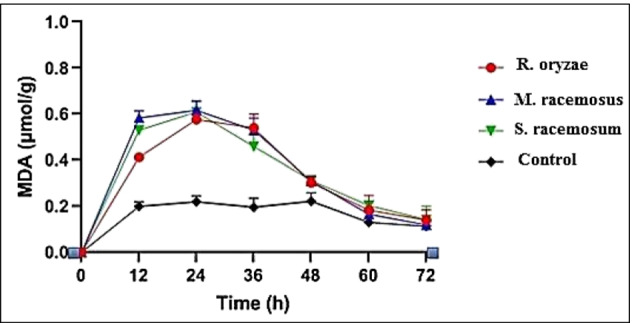
Effect of EACE *A. terreus* on the level of MDA of *R. oryzae*, *M. racemosus*, *and S. racemosum* at different times

We further estimated the MDA level of all tested fungal hyphae because cell membrane phospholipids were a major target of reactive oxygen species (ROS) where excess radical species produce significant change and modification in cell component (Du et al. 2020). The cell membrane lipid peroxidation will irreversibly damage the structural integrity, leading to the rise of membrane permeability and the leakage of intracellular constituent from the cells [44]. Therefore, we also evaluated the structural integrity of the cell membrane. As we expected, treatments of EACE at MIC on three tested fungal strains significantly improved the MDA level up to 24 h from the time of incubation as compared with control, and then a slight decrease in MDA level was observed after 36 h (Fig. 4), while the MDA level the control remained mostly unchanged. Our findings support a mechanism of action in which the studied extract changes the lipid bilayer of fungal membranes first. From a biochemical aspect, the investigated extract would trigger lipid peroxidation in the fungal membrane, according to the existing findings. Increased MDA levels in membrane unsaturation in more susceptible fungi suggest a mechanism involving cellular fatty acid double bonds and, specifically, lipid peroxidation. MDA was used as a stable biochemical marker to see if the fungus under investigation was involved in lipid peroxidation [45]. These results indicated that the treatments of EACE *A. terreus* indeed resulted in the membrane lipid peroxidation of the hypha and damaged the structural integrity of the cell membrane. These indicated that EACE *A. terreus* has a promising antifungal activity against fungi causing mucormycosis.

### Ultrastructure of Fungal Cells

**Fig. 5:**
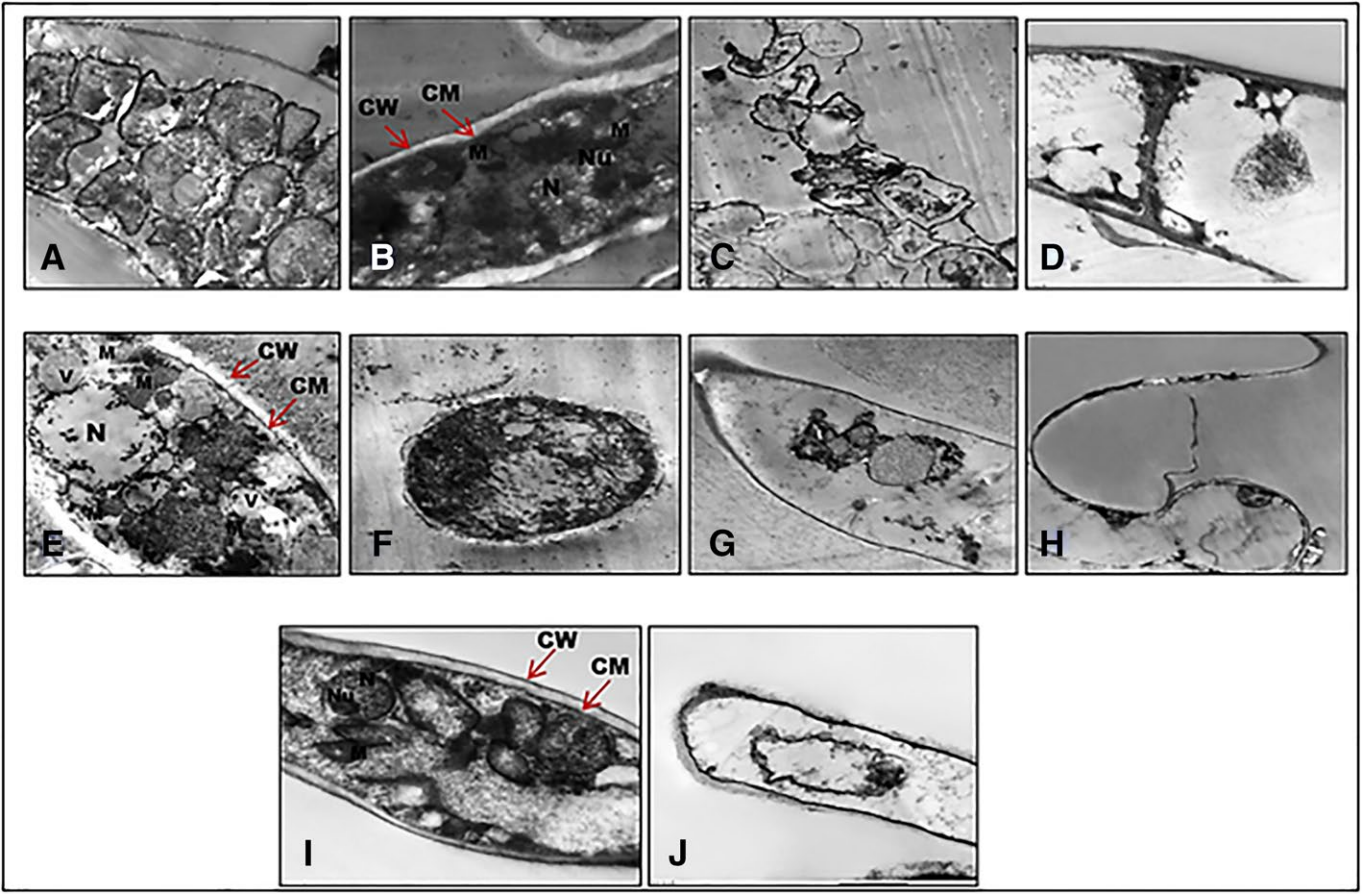
Transmission electron micrographs of *R. oryzae*, *M. racemosus*, *and S. racemosum*. **A** and **B** untreated *R. oryzae*. **A** sporangium and **B** hyphae with the normal ultra-structural components; **C** and **D** treated *R. oryzae* with irreversible ultra-structural changes; **E** and **F** *Mucor*; **E** control sporangium and **F** hyphae; **G** and **H** treated *M. racemosus*. **I** and **J**, **I** control of *S. racemosum* and **J** treated *S. racemosum*. CW, cell wall; CM, cell membrane; M, mitochondrion; V, vacuole; N, nucleus; Nu, nucleolus

The ultrastructural alterations of the studied fungi were determined using transmission electron microscopy (TEM). The ultrastructure of all three species studied *R. oryzae*, *M. racemosus*, *and S. racemosum* was affected by EACE of *A. terreus*. In contrast to the controls, the micrographs of the treated *R. oryzae* found that EACE of *A. terreus* causes permanent ultrastructural alterations in both sporangium and hyphal structures (Fig. 5C and D); the organelles had been destroyed and there is a high leakage of cytoplasmic matrix; the whole cytoplasmic (cytosol) material was observed. *M. racemosus* affected with EACE represented completely deformations of both cell wall and cell membrane with disappearance of the cytoplasmic materials (Fig. 5G and H). Also, the affected *S. racemosum* showed the external layer of the cell wall was thin and electrodense, while the thick inner wall was less and uniform electrodense. The cell membrane was tightly adhered to the cell wall and the cytoplasmic materials constricting in the center of the cell (Figure J). While, control micrographs (without treatments); with well-defined ultrastructure organelles; intact cell wall (CW), cellmmembrane (CM), Mitochondria (M), vacuole (V), nucleus (N) and nucleolus (Nu); sporangium and mycelium of *R.oryzae* (Figs. 5A and B); *M. racemosus* (Fig. 5E and F) and *S. racemosum* (Fig. 5G).

## Conclusion

In this study, endophytic *A. terreus* was isolated from healthy *Moringa oleifera* leaves and identified morphologically and genetically. GC–MS results of EACE of *A. terreus* revealed that fungal extract contains 16 major bioactive compounds with extensive pharmaceutical activities. The EACE *A. terreus* has antifungal activity toward fungi causing mucormycosis. The EACE of *A. terreus* affects disturbance in cell membranes of the tested fungal strains where lipid peroxidation of membranes was elevated. Moreover, ultrastructure changes were observed which confirmed the leakage of cell membrane and cytoplasm. Eventually, the endophytic *A. terreus* is promising for producing biological compounds which can be used for inhibition of mucormycosis fungi.

## Data Availability

The datasets generated during and/or analyzed during the current study are available from the corresponding author on reasonable request.
